# Abnormal Growth and Feeding Behavior Persist After Removal of Upper Airway Obstruction in Juvenile Rats

**DOI:** 10.1038/s41598-017-02843-5

**Published:** 2017-06-02

**Authors:** Mohammad H. Assadi, Elena Shknevsky, Yael Segev, Ariel Tarasiuk

**Affiliations:** 1 Sleep-Wake Disorders Unit, Soroka University Medical Center, P.O. Box 151, Beer-Sheva, 84105 Israel; 2 Department of Physiology, Faculty of Health Sciences, Ben-Gurion University of the Negev, P.O. Box 105, Beer-Sheva, 84105 Israel; 3 Shraga Segal Department of Microbiology and Immunology, Ben-Gurion University of the Negev, P.O. Box 105, Beer-Sheva, 84105 Israel

## Abstract

Pediatric obstructive sleep-disordered breathing is associated with growth retardation, but also with obesity that has a tendency to persist following treatment. We investigated the effect of upper airways obstruction (AO) and of obstruction removal (OR) in juvenile rats on gut-derived ghrelin and related hypothalamic factors, feeding, and growth hormone (GH) homeostasis. Here, we show that after seven weeks of AO, animals gained less weight compared to controls, despite an increase in food intake due to elevated ghrelin and hypothalamic feeding factors. OR rats who had complete restoration of tracheal diameter, consumed more food due to increased ghrelin and exhibited growth retardation due to deregulation of GH homeostasis. This study is the first to show dysregulation of the hormonal axes controlling feeding behavior and growth that are not fully restored following OR. Thus, surgical treatment by itself may not be sufficient to prevent post-surgical increased food intake and growth retardation.

## Introduction

Sleep-disordered breathing (SDB) in adults elicits a cascade of complex endocrine derangements that cause sleep abnormalities, increased energy intake, and obesity^1^. Pediatric SDB has been shown to cause insufficient body weight gain and growth retardation while, in some studies, metabolic syndrome and obesity were observed^2–7^. Interestingly, SDB treatment by adenotonsillectomy (usually the first line treatment in children) is frequently associated with weight gain, thus increasing the risk for obesity, despite normalization of sleep and respiration^2, 3, 6^. It has been proposed that a shift toward sedentary lifestyles and high caloric food choices are predominantly responsible for this weight gain^1–3^.

We have previously shown that the juvenile upper airway obstruction (AO) rat model mimics many of the features of pediatric SDB including sleep fragmentation and growth retardation^8–10^. To the best of our knowledge, the role of the appetite-related neuroendocrine factors on eating behavior in AO and after obstruction removal (OR) have not been well-characterized. Abnormal growth hormone (GH)/insulin-like growth factor 1 (IGF-1) homeostasis^7, 11^ and appetite-related factors such as ghrelin and leptin^12–14^ can play a role in AO-associated insufficient body weight gain. Growth hormone and circulatory liver-derived IGF-1 have major effects on lipid metabolism and body composition^15–17^. Feeding is regulated by the gut-derived hormone ghrelin, and by the hypothalamic release of orexin and ghrelin^18–21^. Orexin plays an important integrative link between control of ventilation and homeostatic challenges such as sleep and feeding^22–25^. Released from the oxyntic glands of the stomach in response to fasting, ghrelin stimulates feeding and release of growth hormone releasing hormone (GHRH) from the hypothalamus^18, 21^ by activation of the GH secretagogue receptors (GHSR)^26, 27^. Neuropeptide Y (NPY), agouti-related peptide (AgRP), and GHRH neurons all express GHSR and are well-characterized targets for ghrelin action on food intake^28, 29^. However, skeletal growth acceleration with ghrelin is dose- and pattern-dependent^30–32^. Short exposure to ghrelin stimulates GH by activating the hypothalamic GHRH via GHSR. Prolonged exposure to ghrelin leads to desensitization of the axis and further ghrelin treatment response.

Intermittent hypoxia associated with SDB can lead to liver injury and to increased risk for obesity^33–35^. The most characterized cellular adaptive response to acute and chronic hypoxia is up regulation of hypoxia-inducible factors (HIFs). Under normoxic conditions HIFs undergo protein hydroxylation and degradation by prolyl hydroxylases (PHDs). In hypoxia, PHDs are suppressed and cellular HIFs are stabilized, improving cell survival^36^. Although no noticeable blood gas exchange abnormalities are evident in AO animals^8–10, 37, 38^, it is unclear if this condition is associated with liver oxidant stress or liver injury.

The mechanisms linking upper airway obstruction with abnormal energy metabolism and growth retardation are poorly understood. We hypothesized that AO leads to sustained elevation of gut-derived ghrelin, which, on one hand, causes desensitization of the hypothalamic-pituitary-GH axis while, on the other hand, increases feeding behavior. In this study we explored for the first time, to the best of our knowledge, the pathophysiological consequences of upper airway loading and of its removal on feeding behavior, linear growth, and endocrine pathways from weaning to adulthood. We show that AO, even long after the critical tracheal obstruction is removed, leads to persistent dysregulation of hormonal axes controlling feeding and growth. Increased feeding after AO, whether treated or untreated, is related to elevated gut-derived ghrelin hormone and its hypothalamic factors and to up regulation of hypothalamic orexin. Our evidence, therefore, indicates that upper airway obstruction elicits persistent neurohumoral derangements that are “stamped” on the hypothalamic pituitary axis of feeding and growth. Our data suggest that the surgical treatment *per se* may not be sufficient to prevent the post-surgical trend for increase in body weight and growth retardation.

## Results

### Food intake and body weight

**Figure 1:**
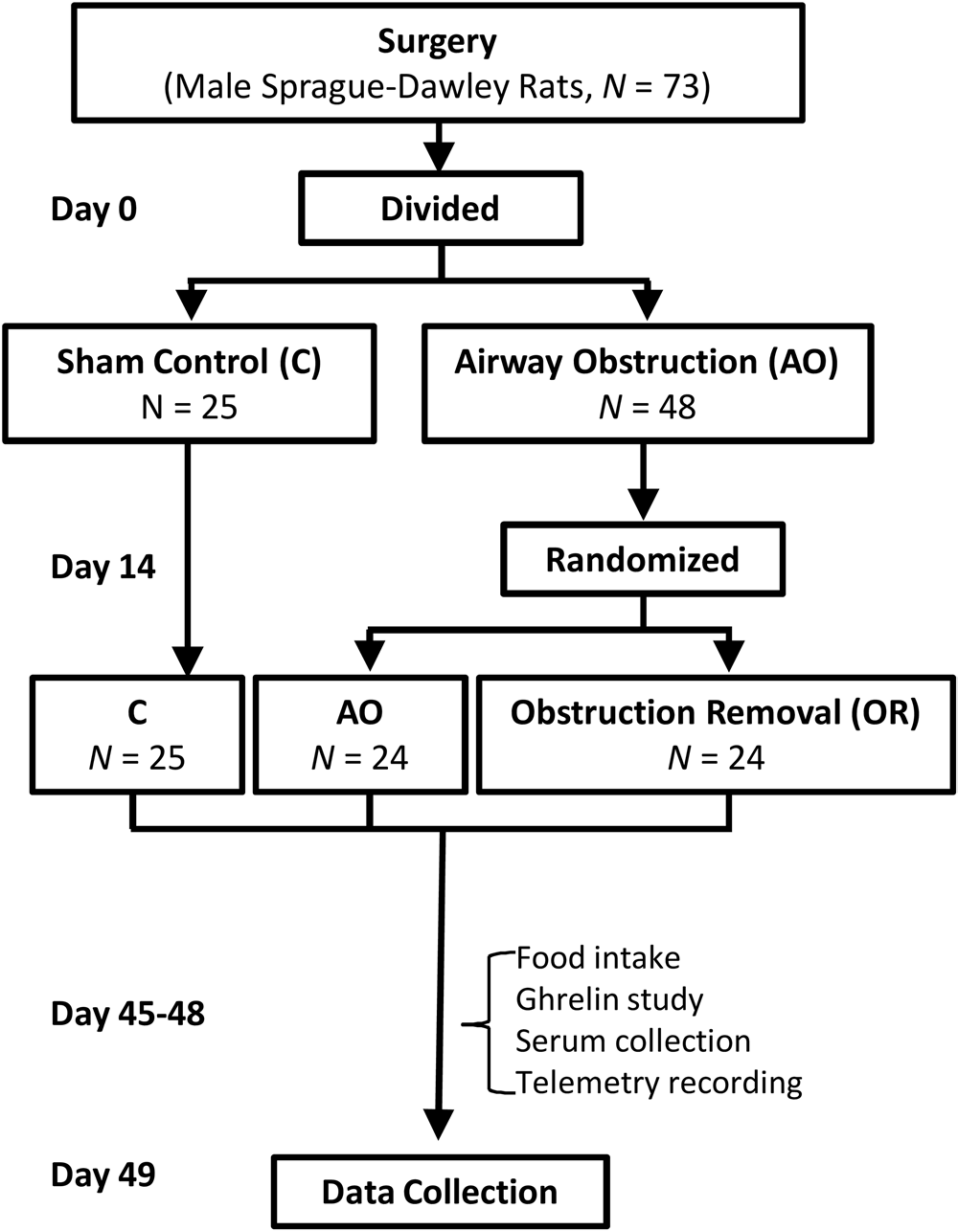
Flow diagram of study groups and times data was collected.

**Figure 2:**
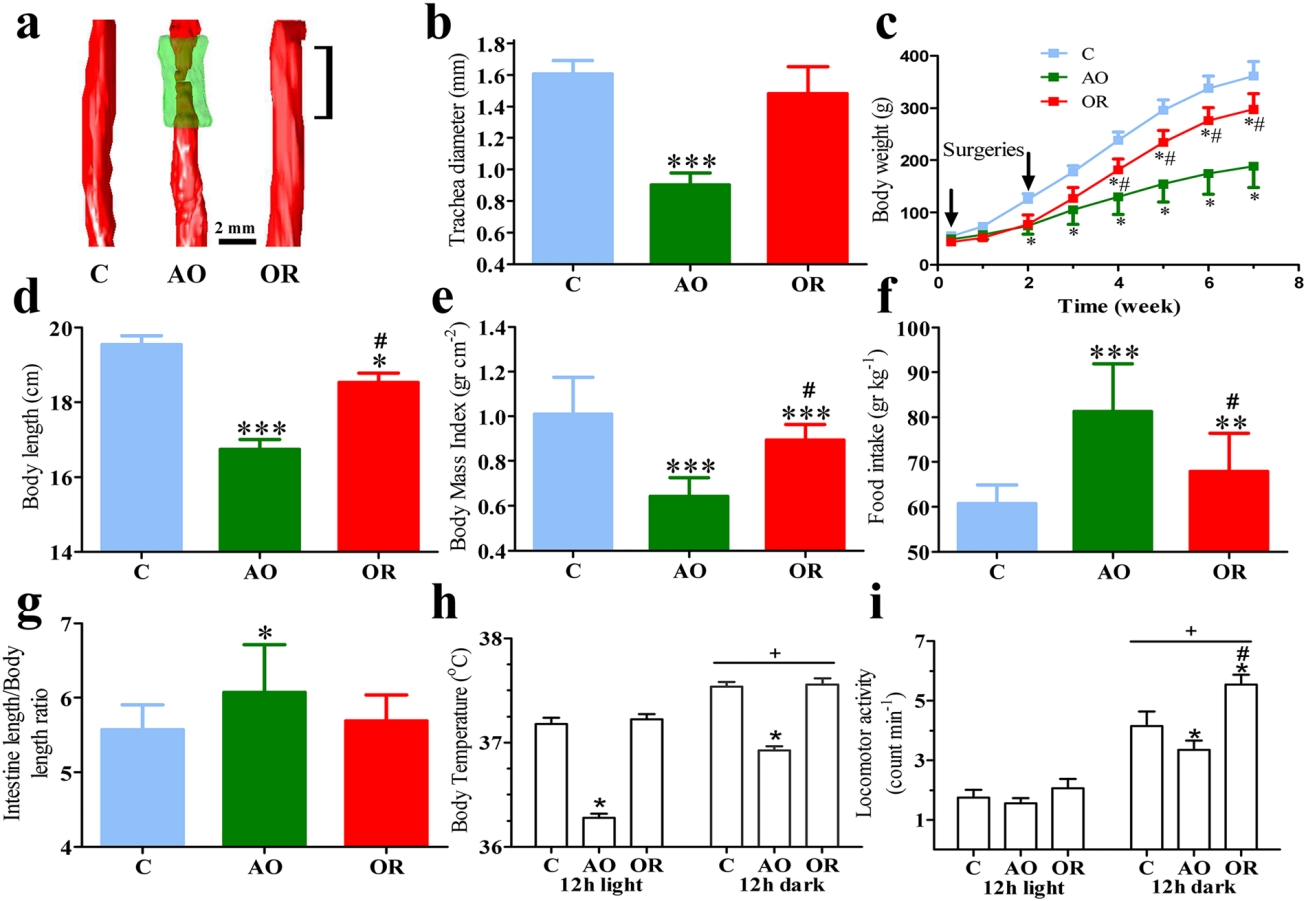
Body weight and daily food intake. Effect obstruction (AO) and obstruction removal (OR) on (**a**) Trachea 3D magnetic resonance imaging (symbol on OR image indicates the location of obstruction); (**b**) Trachea diameter; (**c**) Body weight curve over the duration of the observation period; (**d**) Body length; (**e**) Body mass index; (**f**) Daily food intake; (**g**) Intestinal length to body length ratio; (**h**) 12-hour average lights on and lights off body temperature and locomotion activity (**i**). Error bars in (**b**,**d**,**h**,**i**) are s.e.m; error bars in (**c**,**e**,**f**,**g**) are s.d. *p < 0.05, **p < 0.01, ***p < 0.001 difference between control group (C, blue color) and AO group (green color). ^#^ *p* < 0.05 difference between AO and OR (red color). In (**b**), *d–i* statistical differences were determined by unpaired 2-tailed t test; In (**c**) statistical difference between groups were determined by two-way ANOVA followed by Student-Newman-Keuls test.

**Figure 3:**
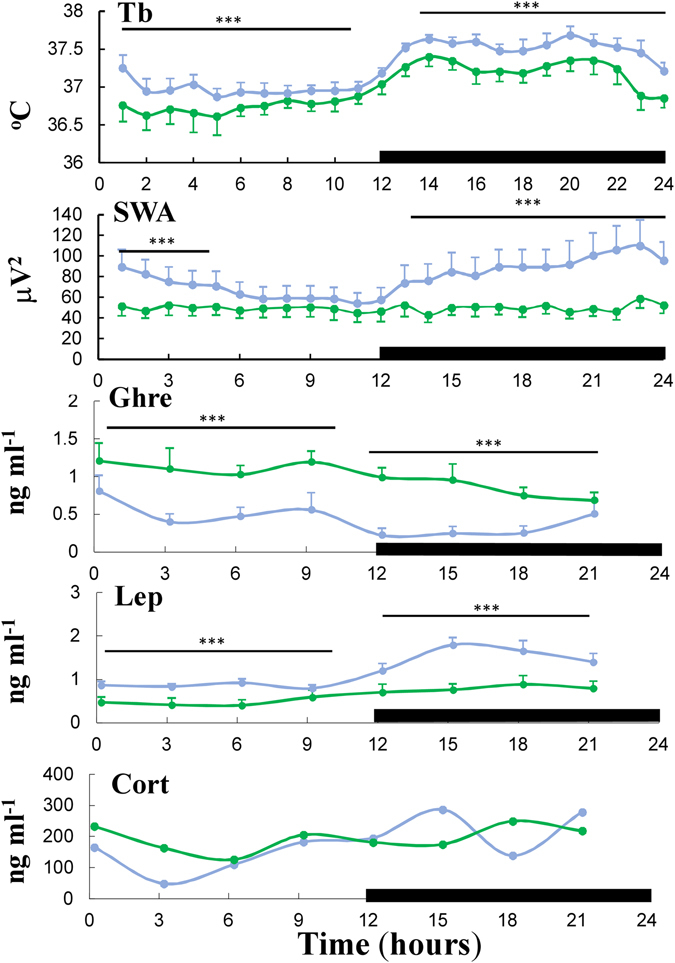
Diurnal rhythms of body temperature, sleep, and hormones in freely moving conditions. Diurnal rhythms of body temperature (Tb) and electroencephalogram slow wave activity (SWA; values are hourly average ± SEM); plasma ghrelin (Ghre), plasma leptin (Lep), and plasma corticosterone (Cort); sera for hormone determinations were collected every 3 hours (mean ± SEM) in freely moving conditions; black horizontal bars represent lights off (active period, 21:00–09:00) on a 12:12-h cycle. Error bars are s.e.m. ***p < 0.001 difference between control group (blue color) and obstructive group (green color). Differences between groups were determined by two-way ANOVA followed by Student-Newman-Keuls test.

Figure 1 illustrates the time-line of data collection. According to magnetic resonance imaging analysis, following AO the trachea diameter was reduced by 44% (*p* < 0.001) and after OR it was 13% (n.s.) below control values (Fig. 2a,b). Trachea cross-sectional area according to histology analysis was reduced by 70% (*p* < 0.001) in AO; after OR trachea cross-sectional area was 20% (*p* < 0.05) smaller than in controls (Supplementary Fig. S1). AO gained 48% and 15% less body weight and body length than controls, respectively (*p* < 0.001; Fig. 2c,d), despite 35% elevation of food intake (*p* < 0.01; Fig. 2f). Despite the 12% elevation in food intake in OR, body weight and body length were 24% and 5% less than those of controls, respectively (*p* < 0.001; Fig. 2c,d,f). Body mass index (BMI) was reduced by 36% and 11% in AO and OR groups, respectively (*p* < 0.001; Fig. 2e). Body temperature (Tb) was lower in the AO group (*p* < 0.05; Fig. 2h) and was similar to controls in the OR group. Dark phase locomotion activity (MA) was reduced by 20% and increased by 33% in AO and OR groups, respectively (*p* < 0.05; Fig. 2i). Arterial blood gases were within physiological range in all groups (Supplementary Table S1). No histological evidence for hepatic damage such as pericellular fibrosis (Supplementary Fig. S2A) or steatosis (Supplementary Fig. S2B) was found. Liver enzymes were within physiological range in all groups (Supplementary Table S1). No changes were found in liver PHD2 mRNA and protein levels (Supplementary Fig. S2C,D). Liver HIF1α mRNA expression decreased (*p* < 0.001; Fig. 3e) and no change was found in HIF 1α protein level (Supplementary Fig. S2F). Liver HIF2α mRNA expression was similar in all groups (Supplementary Fig. S2G) while HIF2α protein level was undetectable using Western blot analysis.

### Diurnal rhythms of sleep and hormones

Mean Tb for each hour of the day is presented in Fig. 3. Both light and dark phase Tb decreased by 0.7 °C in the AO group (*p* < 0.01; Fig. 3). The AO group was awake 20% more during the 12-hour lights on period (*p* < 0.01), and showed 15% less slow wave sleep (*p < *0.05) and 7% less paradoxical sleep (*p* < 0.001). The time course of slow wave activity during slow wave sleep (non-rapid eye movement) of the AO group was 50% lower than in controls, and did not exhibit circadian variation (*p* < 0.001; Fig. 3). No significant differences were found in both diurnal and nocturnal plasma corticosterone level; plasma leptin decreased by 40% in AO (*p* < 0.001; Fig. 3).

### Ghrelin and hypothalamic mediator factors

**Figure 4:**
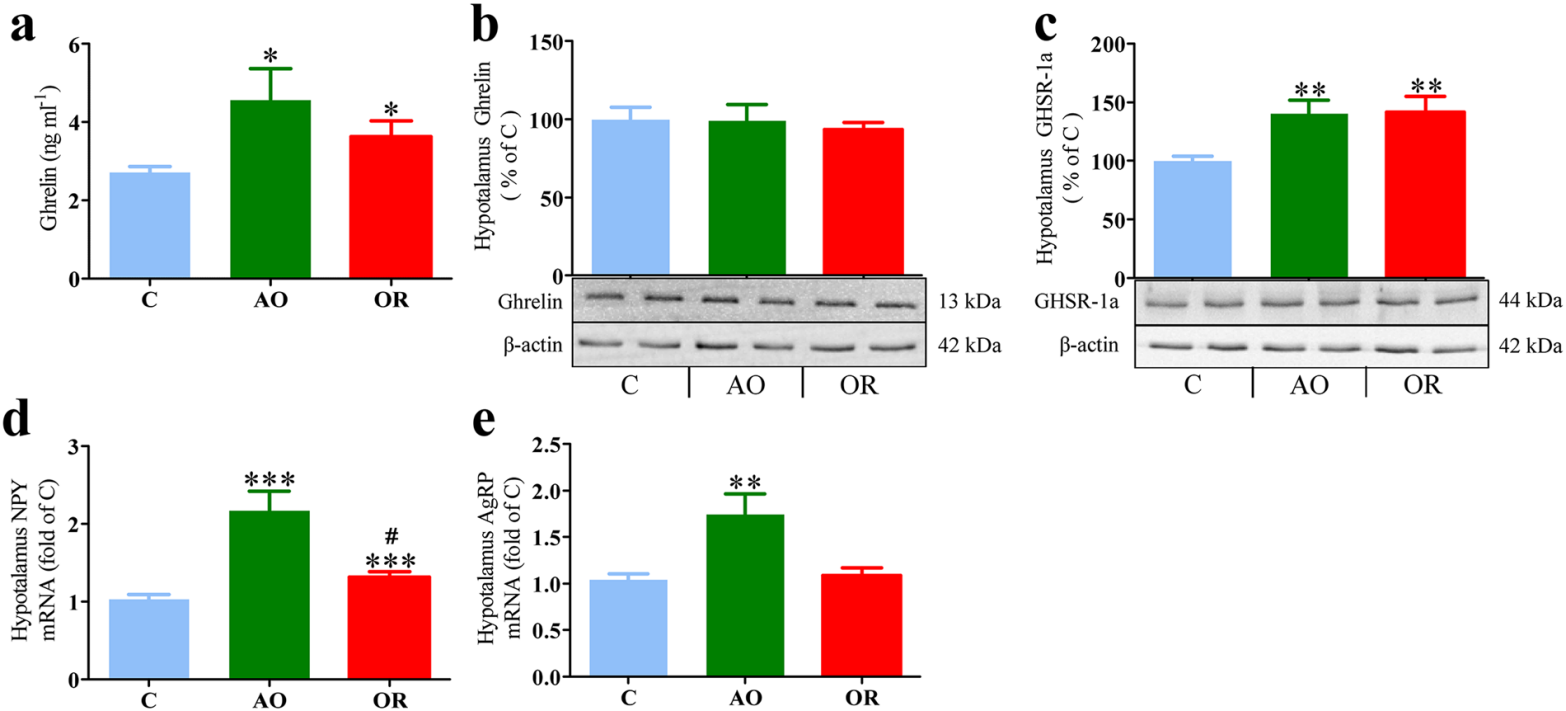
Plasma ghrelin and its related hypothalamic mediator factors. (**a**) Plasma ghrelin; (**b**) Representative hypothalamic ghrelin protein determined by Western blot; (**c**) Representative GHSR1α protein determined by Western blot; (**d**) Hypothalamic NPY relative mRNA level; (**e**) Hypothalamic AgRP relative mRNA level. A densitometric analysis on separate Western immunoblot analyses summarizing the eight animals per group; AgRP – Agouti-related protein; NPY – Neuropeptide Y; GHSR1α – GH secretagogue receptor 1 alpha. Error bars are s.e.m. Statistical differences were determined by unpaired 2-tailed t test. **p < 0.01, difference between control group (C, blue color) and airway obstruction group (AO, green color). ^#^ *p* < 0.05 difference between AO and obstruction removal (OR).

**Figure 5:**
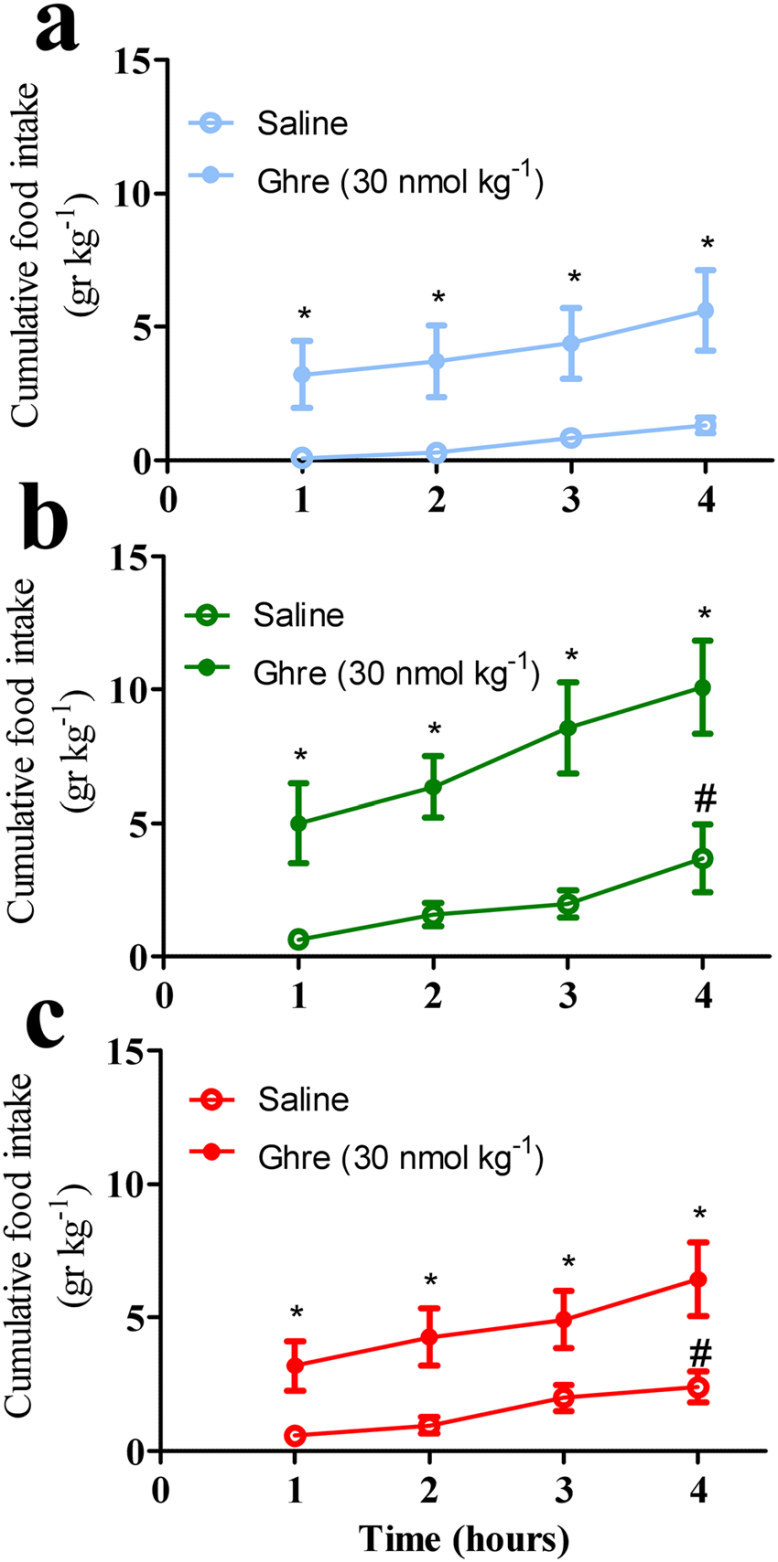
Effect of ghrelin on cumulative food intake. (**a**) Control group; (**b**) Obstructive group; (**c**) Obstruction removal group. Open symbols are mean cumulative 4 h food intake after saline administration and closed symbols follow administration of ghrelin (30 nmol kg^−2^) on the following day. Blue color – control, green color – obstructive group, red color – obstruction removal. Error bars are s.d. *p < 0.05 statistical difference between saline and ghrelin were determined by two-way ANOVA followed by Student-Newman-Keuls test. ^#^ *p* < 0.05 difference between AO and obstruction removal (OR).

Plasma ghrelin was elevated by 200% in the AO group (Fig. 3), and remained 34% higher in the OR group (Fig. 4a). No significant differences were found in hypothalamic ghrelin protein (Fig. 4c). GHRSR1a protein level was up regulated in the AO and OR groups by 40% (*p* < 0.01; Fig. 4c). NPY mRNA expression was elevated by 117% and 32% in AO and OR groups, respectively (*p* < 0.001; Fig. 4d). AgRP mRNA expression was up regulated by 74% in the AO group (*p* < 0.01; Fig. 4e). Injection of ghrelin (30 nmol kg^−1^ i.p.) induced considerable increase of food intake by 300% in all groups (Fig. 5).

### The growth hormone axis

**Figure 6:**
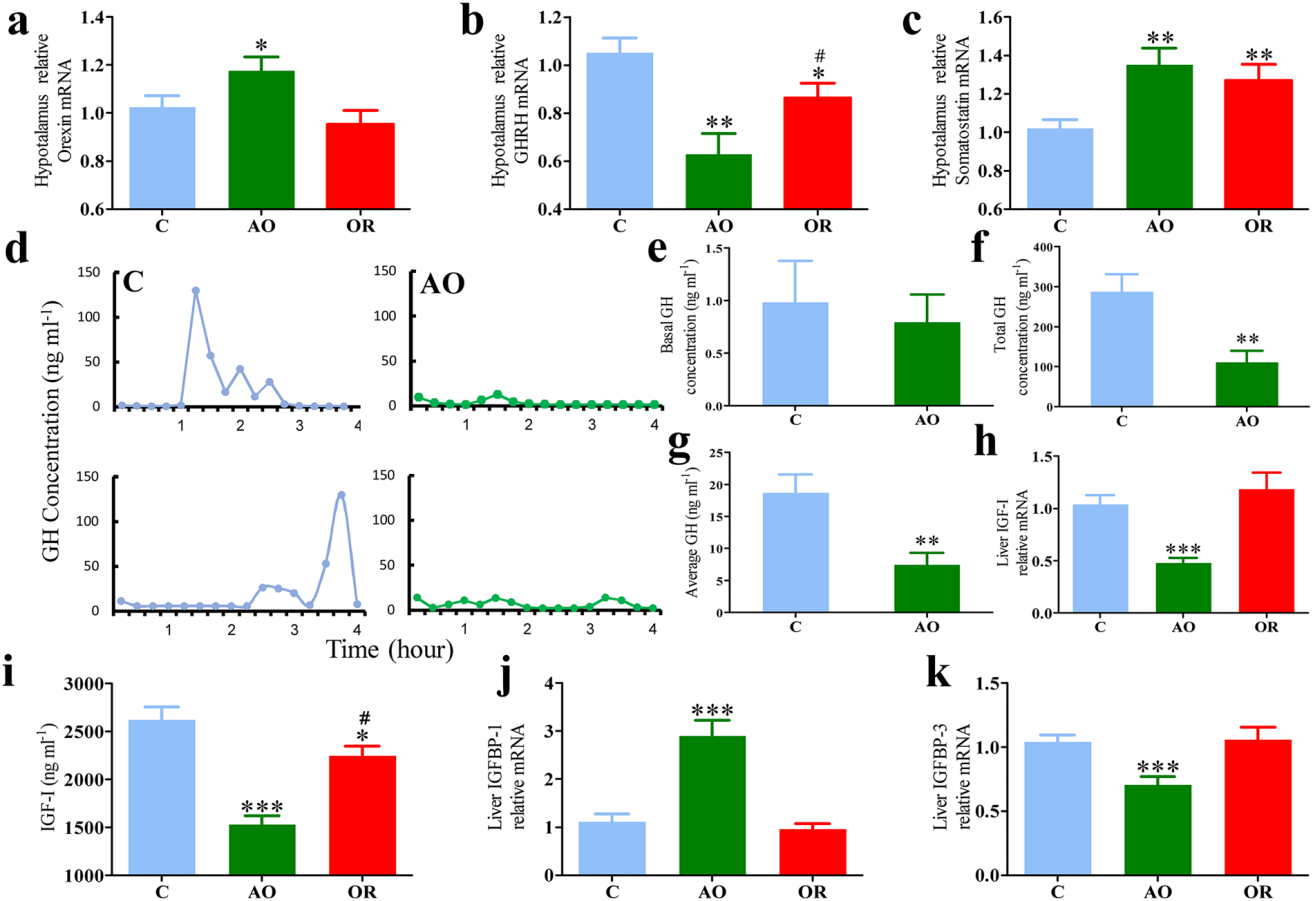
The hypothalamic-pituitary-GH axis. (**a**) Hypothalamic relative orexin mRNA; (**b**) Hypothalamic relative GHRH mRNA; (**c**) hypothalamic relative somatostatin relative mRNA; (**d**) Pulsatile GH concentrations in two representative C and two AO animals; (**e**) average baseline value of GH; (**f**) Total GH concentration; (**g**) Average GH concentration; (**h**) Liver IGF-1 relative mRNA; (**i**) Serum IGF-1; (**j**) Liver IGFBP-1 relative mRNA; (**k**) Liver relative IGFBP-3 mRNA; GH – growth hormone; GHRH – growth hormone releasing hormone; IGF-1 – insulin-like growth factor 1; IGFBP 1 and 3 – IGF-binding protein 1 and 3. Error bars are s.d. *p < 0.05, **p < 0.01, ***p < 0.0001 difference between control group (C, blue color) and obstructive group (AO, green color) or between C and OR obstruction removal (OR, green color). ^#^ *p* < 0.05 difference between AO and OR. Statistical differences were determined by unpaired 2-tailed t test.

After 7 weeks hypothalamic orexin mRNA expression was up regulated by 20% in the AO group (*p* < 0.05; Fig. 6a) and returned to control values in the OR group. GHRH mRNA expression was down regulated by 38% and 14% in the AO and OR groups, respectively (*p* < 0.01 and *p* < 0.05; Fig. 6b). Somatostatin mRNA expression increased by 35% and 27% in AO and OR groups, respectively (*p* < 0.01; Fig. 6c). Basal GH secretion was determined by analysis of blood specimens collected every 15 min during 4-h periods in n = 7 animals in each group. Representative profiles of the GH secretory periods of two control and two AO animals are shown in Fig. 6d. In the control group high amplitude GH peaks (<120 ng ml^−1^) were detected in all animals, and occasional intermediate peaks (i.e., 30–50 ng ml^−1^) were detected in some animals; no AO animals exhibited high-amplitude or pulsed intermediate-amplitude. Most (>90%) GH pulses in the AO group were considered small. Overall, the frequency of pulses determined using *AutoDecon* ^39, 40^ was appreciably different in the AO group. Basal GH secretion (prior GH peaks) was similar in control and AO groups (Fig. 6e). Total and average GH concentration decreased by 60% in the AO group (*p* < 0.001; Fig. 6f,g). Both liver IGF-1 mRNA and protein were down regulated by 52% and 42%, respectively, in the AO group (*p* < 0.001; Fig. 6h,i). Obstruction removal only partially improved IGF-1 protein level, which remained lower than that of the control group by 15% (*p* < 0.05; Fig. 6i). IGFBP-1 mRNA expression was up regulated and IGFBP-3 mRNA expression was down regulated by 190% and 30% in the AO group, respectively (*p* < 0.001; Fig. 6j,k).

## Discussion

This study demonstrated that in upper airway obstruction, there was persistent deregulation of the hormonal axis controlling feeding behavior and growth. This was not reversed following obstruction removal (treatment). Elevation of gut-derived ghrelin and its hypothalamic mediators was associated with hyperphagia and altered hypothalamus-pituitary GH axis due to down-regulation of GHRH and up-regulation of somatostatin.

Little is known about long-term effects of pediatric SDB treatment from childhood to adulthood. To our knowledge this is the first study that explored the effects of AO and its removal on energy metabolism and the hypothalamic-pituitary-GH axis from weaning to adulthood. Upper airway obstruction was performed on 22-day-old rats and animals were followed for two weeks prior to obstruction removal (Fig. 1). This period is comparable to half a year to 6–8 years in children^8^ and this period can lead to abnormal sleep and growth retardation^8, 41, 42^. The peak prevalence of pediatric SDB occurs at two to eight years, which is the age when tonsils and adenoids are the largest in relation to the site of collapse; and adenotonsillectomy is usually first line treatment^4, 6^. Obstruction removal was performed on day 14 and animals were followed for five weeks. This period is roughly comparable to more than fifteen years in humans. Our model has implications for pediatric SDB; similar to sleep apnea, AO animals exhibit abnormal sleep and growth retardation. However, in our study both inspiratory and expiratory loading was induced, which may resemble subglottic stenosis in children and not be exclusively sleep related. We did not find evidence for stress in that serum corticosterone levels were similar to those of controls. Under these conditions, animals maintain their arterial blood gases and serum lactate^43^. Intermittent hypoxia can lead to liver injury^33, 34^. In our study we did not find changes in liver enzymes, liver histology, PHD2, HIF1α and HIF2α (Supplementary Fig. S2). These findings indicate that hypoxia probably does not play a role in this abnormal feeding and growth in AO animals.

Obstructive animals exhibited impressive hyperphagia that was related to elevation of gut-derived ghrelin and the array of mediators that are activated by ghrelin, and decreased circulatory leptin (Fig. 4). Short sleep *per se* can stimulate gut-derived ghrelin and feeding^44^. In our study AO animals were awake 20% more during the 12-hour lights on period. Ghrelin increased signals associated with motivated behaviors of importance for survival such as increased wakefulness, MA, and feeding^18, 45, 46^. Increased feeding in AO is probably a physiological adaptation to provide energy needed to sustain the additional wakefulness^47^ and the increased work of breathing. Although hypothalamic GHSR1α protein increased, it was not related to desensitization of feeding, since administration of ghrelin stimulated food intake in all groups. Feeding could also be stimulated by orexin^22–24^. Enhanced orexin secretion is crucially important for respiratory homeostasis maintenance and partial sleep loss in AO animals^9^. Orexin-A consistently stimulates food intake, while orexin-B only does so occasionally^22, 23, 48^. Orexin and ghrelin-containing neurons could influence each other and thereby regulate feeding behavior^21^. The neuroendocrine effects in AO may resemble those seen following prolonged partial sleep-restriction in rats, including decreased Tb^49^, down regulation of hypothalamic GHRH, up regulation of somatostatin^44, 50^, and reduction of GH^51^.

Interestingly, here we provide evidence of deregulation of hormonal control of feeding long-term following treatment. The OR group continued to display hyperphagia due to increased gut-derived ghrelin and hypothalamic NPY, although trachea diameter, Tb, and hypothalamic orexin mRNA were similar to those of controls. Further studies are needed to explore the whole-body energy demand and energy expenditure using an open-circuit indirect calorimeter^47^, match for the number of calories consumed during the day, and verify protein content of hypothalamic feeding mediators. It is possible that elevated energy intake in OR is met with higher energy expenditure related to increased MA during dark phase (Fig. 2). Adenotonsillectomy has been reported to accelerate the risk for obesity, despite the normalization of sleep and breathing^2, 3^. The increased risk for obesity was attributed to a shift toward sedentary lifestyle and highlights the importance of lifestyle modifications following medical intervention^1–3^. It was recently found in adults that although a reduction in basal metabolic rate after positive airway pressure treatment predisposes to a positive energy balance, dietary intake and eating behavior had greater impacts on weight change and tendency to develop positive body weight gain and obesity^1^. Thus, weight gain following relief of upper airway obstruction could be at least in part related to permanent alterations in appetite homeostasis.

Gut-derived ghrelin plays an important integrative role in stimulating GH and feeding^19, 20, 52^ by activation of GHSR^26^. Short exposure to ghrelin stimulates GH by activating the hypothalamic GHRH^30–32, 46, 53^, while prolonged exposure leads to desensitization of the GH^30–32^. In our study we found prolonged elevation of ghrelin associated with desensitization of the hypothalamic-pituitary-GH axis in the AO group (Fig. 6). This desensitization was related to elevation in hypothalamic somatostatin and reduction of GHRH; both regulate the pulsatile secretion of GH. Activation of orexin neurons can inhibit GHRH in hypothalamic nuclei involved in regulation of sleep and GH^54^. The need to maintain ventilation in AO leads to elevation of hypothalamic orexin, which results in abnormalities in the GHRH/GH axis that underlie both growth retardation and slow wave abnormalities; supporting earlier studies^9, 50^. Circulating IGF-1 and IGF-binding protein 3 (IGFBP-3) strongly correlated with the 24-hour mean GH levels^55, 56^. Our study shows that the decreased GH was followed by downstream reduction of liver IGF-1 and IGFBP-3 and circulatory IGF-1. One of the interesting findings of our study is that GH homeostasis does not return to control values long-term after “treatment” (OR) and animals exhibited shorter body length. This desensitization of the GH axis was accompanied by an elevation in somatostatin and reduction of GHRH. In pediatric SDB, the impaired GH hypothesis has received a great deal of attention lately as a key mechanism underlying impaired somatic growth in these children^7^. Meta-analysis^11^ revealed that standardized height and weight, IGF-1, and IGFBP-3 increase after adenotonsillectomy, lending support to the concept that GH homeostasis is impaired prior to treatment in pediatric SDB. However, these studies used the “before and after” data analysis; healthy controls were not used and children are followed up to a year post-surgery^11^. Further studies are needed to explore the long-term alterations in GH homeostasis following treatment in clinical and animal models.

The mechanisms linking upper airway obstruction with abnormal energy metabolism and growth are poorly understood. To our knowledge, this study is the first to show that upper airway obstruction, even long after the critical tracheal obstruction is removed (treatment), leads to persistent deregulation of the hormonal axis controlling feeding and growth. Our data may suggest remodeling of the central pathways regulating appetite and growth. Increased feeding following upper airway obstruction, whether treated or untreated, is related to elevated gut-derived ghrelin hormone and its hypothalamic mediator’s factors’ (e.g., GHSR1α, NYP, AgRP) levels and to up regulation of hypothalamic orexin. Growth and endocrine markers of feeding do not reach control values for a long period after AO with or without OR. Our evidence, therefore, may indicate that the surgical treatment of pediatric SDB by itself may not be sufficient to prevent the postsurgical trend for increase in body weight and obesity. Restoration of the neuroendocrine system responsible for adequate feeding and growth seems to be essential for postsurgical management.

## Methods

### Animals

This study was approved by the Ben-Gurion University of the Negev Animal Use and Care Committee protocol number: IL-77-11-2015, and complied with the American Physiological Society Guidelines. Male Sprague-Dawley 22-day-old rats (48–55 gr) were used. Animals were kept on 12–12 light-dark cycle with lights on 09:00 at 23 ± 1.0 °C. Food and water were given *ad libitum*.

### Surgery

Tracheal narrowing surgery (anesthesia tribromoethanol, 200 mg kg^−1^ i.p.) was used to induce UAO in 22-day-old Sprague-Dawley male rats (day 0; Fig. 1)^8–10, 57^. Controls underwent surgery with no tracheal narrowing. On day 14 the AO group was randomized; OR of the silicon band was performed on n = 24 animals; all control and AO animals underwent repeat sham surgery. On day 39 a telemetric transmitter (TL11M2-F20-EET Data Sciences International, St. Paul, MN, USA) was implanted (under sterile conditions), enabling recording of electroencephalography (EEG), dorsal neck electromyography (EMG), and body temperature (see Supplementary Methods)^8^. For Tb and MA recording, a free-floating transmitter (model TA11TA-F10, DSI, St. Paul, MN, USA) was inserted into the abdominal cavity on day 40. The transmitter was able to freely move among the peritoneal organs, because it was not attached to the peritoneum^9, 42^. A venous catheter was implanted in the external jugular and advanced to the right atrium for sampling of arterial blood in unrestrained animals on day 36^58^. A 3 F heparin-coated PU catheter (CBAS-C30, Solomon Scientific, San Antonio, TX, USA) was inserted into the common carotid artery, ensuring that the catheter tip was in the thorax. Following surgery, prophylactic enrofloxacin 5 mg ml^−1^ (s.c.) and water containing ibuprofen (0.1 mg ml^−1^) were given for three days^9, 59, 60^.

### Magnetic Resonance Imaging

We used a high performance 1 T compact M2 MRI, 35 mm ID solenoid coil (Aspect Imaging, Shoham, Israel)^10^. Images were acquired (on day 10 and day 20; Fig. 1) with a gradient spin echo sequence, with repetition time (TR)/echo time (TE)/NEX = 13.4/3/2. Multislice axial scans were collected with a 5-cm field-of-view and data matrix of 256 × 256, resulting in 50/256 = 0.195 mm in-plane resolution, slice thickness of 1 mm. Region growing algorithm was used for segmentation of two objects: trachea and the circumferential silicon band used for the trachea narrowing.

### Blood collection

To determine the relationships among plasma appetite-related factors such as ghrelin, leptin, and corticosterone, blood specimens were obtained by repeated sampling of blood every 3 hours for 24 hours, starting at lights on (09:00). Characteristics of pulsatile GH secretion^39, 40^ were performed following a four-day recovery period. In a subset of animals, serum ghrelin and IGF-1 levels were determined at animals’ death, two hours after lights on.

### Telemetry recordings

The duration of sleep-wake states was calculated in 1-h time intervals. These were categorized as: 1) W – wake, 2) SWS – slow wave sleep, and 3) PS – paradoxical sleep (see Supplementary Methods). The power density values for 0.5–4.0 Hz were integrated and used to calculate slow wave activity during non-rapid-eye-movement sleep^8, 42^. Tb ( ± 0.1 °C) and MA were continuously monitored using the Dataquest A.R.T. system (DSI, St. Paul, MN, USA).

### Food intake

To assess the nutritional effect of AO and OR, twenty-four hour food intake was assessed and expressed as grams of food kg^−1^ of body weight^37^.

### Arterial blood gas

In a subset of control, AO and OR arterial blood gases were determined. Blood gas samples of 100 μL were drawn in a pre-heparinized syringe, placed on ice, and immediately analyzed on a blood gas analyzer (RAPID Point 500, Siemens, Erlangen, Germany).

### Serum biochemistry and endocrine

To assess liver function, serum biochemistry was analyzed by the Biochemistry Laboratory of Soroka Medical Center (Beer-Sheva, Israel). Plasma GH, ghrelin, leptin, corticosterone, and serum IGF-1 were determined by specific ELISA kits according to the manufacturer’s instructions. GH, ghrelin, leptin, corticosterone were measured on days 45–48 and IGF-1 after animals’ death on day 49.

### Trachea and Liver Histology

To excise the banded region of the tracheas a similar region from the fifth to the sixth cartilaginous rings of the trachea was excised in the control, AO, and OR groups. Tracheal and liver segments were fixed in 4% formalin for 48 hours at room temperature. After fixation, the silicon band was removed from the tracheal segments of the AO rats. All tracheal segments were then embedded in paraffin, and sections were cut (five μm thickness for the trachea and four μm for the liver) and collected on Superfrost™ Plus slides for histology staining with hematoxylin and eosin^10, 42^.

### Western immunoblot and real time-PCR

Proteins were determined by Western immunoblot^9, 42^ on day 49. RNA extraction and real time-PCR: RNA was extracted, and quantitative real time PCR assays were performed (see Supplementary Methods)^10, 42^. All primers were purchased from Sigma-Aldrich, Rehovot, Israel (Supplementary Table S2). GHRH, Somatostatin, and Orexin primers were used to assess the hypothalamic GH axis. GHSR1α, somatostatin, NPY, AgRP, and β-Actin primers were used to assess hypothalamic ghrelin and related factors. IGF-I, IGFBP-1, and IGFBP-3 primers were used to explore liver growth mediators. PHD2, HIF 1α, and HIF 2α primers were used to assess liver hypoxia inducible factors.

### Experimental schedule

AO or sham control surgery was performed on 22-day-old rats (day = 0) and animals were followed for 7 weeks. Obstruction removal was performed on day 14 on n = 24 randomly selected AO animals. Body weight was measured weekly, MRI images were acquired on day 10 and day 20. Body mass index was calculated by dividing body weight (grams) by body length squared (centimeters squared). Sleep, Tb, and MA were recorded for 24 hrs, and food intakes were measured on days 45–48. Plasma ghrelin, leptin, and corticosterone were determined on day 40 every 3 hours starting at lights in freely moving conditions. GH was determined on day 42 starting at lights onset and blood was collected every 15 minutes for 4 hours in freely moving conditions. Immediately before animal death on day 49 body length was measured and BMI (gr cm^−2^) was calculated. Tissues and serum were harvested between 1 and 2 hours after light onset and were frozen at −80 °C until analysis.

### Data analysis

Two-way analysis of variance for repeated measures was used to determine significance between time and group using post hoc comparisons by Student–Newman–Keuls test. Significance between groups was analyzed by unpaired Student’s *t*-test. Characteristics of GH pulsatile secretion were analyzed with the *AutoDecon* statistical algorithm to separate small peaks from background variation, according to guidelines described by Johnson^39, 40^. Pulses of GH detected by *AutoDecon* additionally were assessed for frequency in those instances in which two peaks were captured during the 4-h sampling period. Null hypotheses were rejected at the 5% level.

## Electronic supplementary material

Supplrmentary information
