# Supplementary material for: Abnormal Growth and Feeding Behavior Persist After Removal of Upper Airway Obstruction in Juvenile Rats
Source: Sci Rep. 2017 Jun 2;7:2730. doi: 10.1038/s41598-017-02843-5 (PMC5457418; doi:10.1038/s41598-017-02843-5)
Supplement: Supplementary file 1 — Supplrmentary information [file 41598_2017_2843_MOESM1_ESM.doc]

# Supplementary Information

**Abnormal Growth and Feeding Behavior Persist After Removal of Upper Airway Obstruction in Juvenile Rats**

Mohammad H. Assadi, Elena Shknevsky, Yael Segev, Ariel Tarasiuk

**Supplementary methods**

**Surgery**

The technique used for sham surgery and to induce AO in 22-day-old male rats was as previously described.^S1-S3^ Animals were anesthetized with tribromoethanol (200 mg kg^-1^) administered intraperitoneally (i.p.). A midline ventral cervical incision was made, and the trachea exposed and dissected so as not to damage adjacent structures. A circumferential silicon band 0.5 cm long was placed around the trachea to induce tracheal-narrowing. Controls underwent sham surgery with no tracheal narrowing. On day 14 the AO group was randomized and obstruction removal (OR) of the silicon band was performed on n=24 animals; the remaining AO animals underwent a second sham surgery. On day 35 after AO/sham surgery a telemetric transmitter (TL11M2-F20-EET Data Sciences International, St. Paul, MN, USA) was implanted (under sterile conditions), enabling recording of electroencephalography (EEG), dorsal neck electromyography (EMG), and body temperature. Leads from the electrodes for EEG recording were placed over the frontal (1.1 mm anterior and 1.1 mm lateral to the bregma) and parietal (3 mm posterior and 1.5 mm lateral to the bregma) cortices. EEG electrodes were anchored to the skull with dental cement.^S2^ For Tb and MA recording, a free-floating transmitter (model TA10TA-F20, DSI, St. Paul, MN, USA) was inserted into the abdominal cavity on day 36. The transmitter was able to freely move among the peritoneal organs, because it was not attached to the peritoneum. The peritoneal muscle and skin layers were closed with interrupted sutures.^S3,S4^ A venous catheter was implanted in the external jugular and advanced to the right atrium for sampling of arterial blood in unrestrained animals.^S5^ A 3F heparin-coated PU catheter (CBAS-C30, Solomon Scientific, San Antonio, TX, USA) was inserted into the common carotid artery, ensuring that the catheter tip was in the thorax. The catheter tubing was extended about 40 cm and was attached to a swivel commutator and counterbalanced boom assembly.^S6^

**Blood collection**

Blood samples were drawn, placed in a microcentrifuge tube on ice, and specimens were centrifuged cold (8,000 *g* for 10 min). The plasma was pipetted into chilled microcentrifuge tubes for subsequent storage at -80°C. To help prevent anemia, the red blood cells were resuspended with heparinized saline, injected through the catheter, and flushed through with heparinized saline.^S6^ Basal pulsatile GH was assessed. Blood was collected every 15 minutes for 4 hours starting at lights on.

**Telemetry recordings**

Raw electroencephalogram (EEG) and electromyography (EMG) outputs from the skull and skeletal muscle electrodes were sampled at 256 Hz, filtered at 0.1–40 Hz and 10–300 Hz, respectively, using the DSI system (DSI, St. Paul, MN, USA).^S2^ The vigilance states were scored using DSI NeuroScore v. 2.1 software and were edited visually for 10-sec epochs on the basis of the predominant state within the epoch.^S2,S7,S8^ Tb (±0.1°C) and MA were continuously monitored using the Dataquest A.R.T. system (DSI, St. Paul, MN, USA). The signal emitted by the transmitter is proportional to Tb. MA (counts) is obtained by counting the number of impulses, detected by changes in signal strength, per unit time. The signal is received by an antenna under each animal's cage and transferred to a peripheral processor connected to a personal computer. All transmitters were calibrated before surgery and at the completion of experimentation to ensure validity of biotelemetry measurements. Tb and MA raw data were collected at 1-sec intervals in unrestrained rats. Raw Tb and MA data are graphically presented as 1-h averages for ease of presentation.^S3^

**Food intake**

Animals were given 40 gr day^-1^ (>40% of maximal daily food intake) of standard rodent chow (Harlan, Jerusalem, Israel). Calories were provided by protein (29.9%), fat (13.4%), and carbohydrate (56.7%) with 13% moisture; energy 3.95 (Kcal kg^-1^). Food was placed into the feeder at the beginning and any remaining at the end of each 24-hr period was weighed. Any visible food in the cage was scavenged and included in the measurements.^S9^

**Serum biochemistry and endocrine**

The low detection limit for GH was <0.5 ng/ml (KRC5311, Thermo Fisher Scientific Inc, Rehovot, Israel) and the intra- and inter-assay CVs were 4.8% and 5%, respectively. The low and high detection limits for leptin were 62.5 and 4000 pg/ml (R&D Systems, Minneapolis, MN, USA), and the intra- and inter-assay CVs were <4% and 6%, respectively. The low and high detection limits for ghrelin were 0.04 and 10 ng/mL (Merck Millipore, Rosh Haayin, Israel); the intra- and inter-assay CVs were 1.1% and 3.2%, respectively. The low and high detection limits for corticosterone were 0.3 and 100 ng/mL (Assaypro, Charles, MO, USA) and the intra- and inter-assay CVs were <5% and 6%, respectively. The low and high detection limits for IGF-I were 25 and 3000 ng mL^-1^; intra- and inter-coefficient of variation (CV) was 4.8% (Diagnostic System Laboratories Inc. Webster, TX, USA). Values that fell below or above the range of assay detection were set equal to the lower or upper limit of sensitivity, respectively.

**Western immunoblot analysis**

Hypothalamus tissue was homogenized on ice with a polytron (Kinetica, Littau, Switzerland) in lysis buffer (50mM Tris, pH 7.4, 0.2% Triton X-100) containing 20mM sodium pyrophosphate, 100mM NaF, 4mM EGTA, 4mM Na3VO4, 2mM PMSF, 0.25% aprotinin, and 0.02 mg/mL leupeptin. Extracts were centrifuged for 20 minutes at 17,000 g at 4°C and the supernatants collected and frozen. Homogenates were mixed with 4× sample buffer and boiled for 5 minutes. Then, 100 μg portions of sample protein were loaded in each gel lane and subjected to 7.5–15% SDS polyacrylamide gel, and electroblotted into nitrocellulose membranes. Blots were blocked for 1 hour in TBST (0.05% Twin-20) buffer (10mM Tris, pH 7.4, 138mM NaCl) containing 5% non-fat dehydrated milk, followed by overnight incubation with primary antibodies such as Ghrelin, GHSR 1α, and HIF 1α (Santa Cruz Biotechnology, Santa Cruz, CA, USA), PHD2 (Bioss Antibodies, Woburn, MA, USA), and β-actin (MP Biomedicals, Solon, OH, USA). After washing 3 times for 15 minutes in TBST (0.05% Twin-20), the blots were incubated with a secondary anti-mouse (β-actin), anti-rabbit (GHSR 1α and PHD2), or anti-goat (Ghrelin) antibodies conjugated to horseradish peroxidase for 1 hour at room temperature and then washed again 3 times. The band antibody was visualized by enhanced chemiluminescence (ECL; Biological Industries, Beit Haemek, Israel) and images were taken using Microchemi 4.2 (DNR Bio image system, Jerusalem, Israel). Densitometric analyses were performed using Image J.^S3,S10^

## RNA extraction and real time-PCR

## Total RNA was extracted from the hypothalamus using the PerfectPure RNA Tissue kit (5 PRIME, Hamburg, Germany); cDNAs were synthesized using high-capacity cDNA reverse transcription kit (Quanta Biosciences, Beverly, MA, USA). Quantitative real time PCR (qPCR) assays were performed with power SYBR green PCR master mix (Quanta Biosciences) using the ABI Prism 7300 Sequence detection System (Applied Biosystems, Foster City, CA). Each sample was analyzed in triplicate in individual assays. The specificity of the reaction is given by the detection of the melting temperatures (Tms) of the amplification products immediately after the last reaction cycle. The target genes expression value was calculated by the ΔΔct method after normalization with a housekeeping gene (β-Actin).^S2,S3^

## Trachea and Liver Histology

Photomicrographs were obtained by light microscope (Olympus BX41, Japan) at ×20 magnification (Zeiss Axioplan MR12, Germany) equipped with a digital camera (Olympus DP72) connected to a PC that used tissue histology morphometric software (CellSens Entry Imaging Software, Olympus(. The internal border of the trachea was outlined and cross-sectional area was calculated for each animal.

**Supplementary Fig. S1:** (A) Representative image of tracheas in control, (B) obstruction, and (C) obstruction removal rats. Tracheas were stained with hematoxylin–eosin, magnification ×20, n = 5 in each group, scale bars = 500 μM. (D) Trachea diameter, error bars are s.e.m.

**** p*<0.001 difference between control group (C, blue color) and AO group (green color).

# *p*<0.05 difference between AO and OR (red color)

**Supplementary Figure S2**: (A) Representative image of liver in control (C), obstruction (AO), and obstruction removal (OR) rats without inflammation (hematoxylin–eosin staining, magnification ×100); (B) Representative image of liver without pericellular fibrosis in all groups (Masson trichrome staining, magnification ×100); (C) Liver PHD2 relative mRNA level; (D) Representative PHD2 protein determined by Western blot; (E) Liver HIF1a relative mRNA level; (F) Representative HIF1a protein determined by Western blot. (G) Liver HIF2α relative mRNA level.

A densitometric analysis on separate Western immunoblot analyses summarizing the n = 8 animals per group. PHD2 – prolyl hydroxylase 2; HIF1α – hypoxia-inducible factor 1-alpha; HIF2α – hypoxia-inducible factor 2-alpha.

Error bars are s.e.m. Scale bars = 100 μM.

**** p*<0.001, difference between C (Blue color) and AO (green color).

Statistical differences were determined by unpaired 2-tailed t test.

**Supplementary Table S1: Arterial blood gases and serum levels of liver enzymes.**

|  | Control | Obstructive | Obstruction Removal |
| --- | --- | --- | --- |
| PO_2_ (mmHg) | 87.3±12.05 | 93.7±8.14 | 84.8±8.9 |
| PCO_2_ (mmHg) | 45.3±5.7 | 41±4.3 | 46.9±6.6 |
| pH (units) | 7.35±0.04 | 7.35±0.05 | 7.34±0.04 |
| HCO_3_^-^ (mEq L^-1^) | 24.5±2 | 23.1±2.3 | 25.8±2.5 |
| ALT (unit L^-1^) | 56±9.5 | 78.7±14.6* | 55.5±6 |
| AST (unit L^-1^) | 117.5±37 | 113.1±35 | 94.3±15.9 |
| Alkaline phosphatase (unit L^-1^) | 299±82 | 340±38 | 301±61 |
| Total bilirubin (mg dL^-1^) | 0.08±0.03 | 0.12±0.03 | 0.09±0.02 |
| Direct bilirubin (mg dL^-1^) | 0.02±0.007 | 0.02±0.014 | 0.01±0.005 |
| Total protein (gr dL^-1^) | 5.74±0.2 | 5.68±0.26 | 5.7±0.17 |
| Albumin (gr dL^-1^) | 2.95±0.1 | 2.94±0.1 | 2.9±0.09 |

PO_2_ – arterial O_2_ pressure; PCO_2_ – arterial CO_2_ pressure; pH – arterial pH; HCO_3_^-^ – calculated arterial bicarbonate; ALT – alanine aminotransferase; AST – aspartate aminotransferase; n = 6 in each group for arterial blood gases; n = 9 for serum levels of liver enzymes; Values are mean ± SD.

* *p*<0.01*; p* value was determined by unpaired 2-tailed t-test.

**Supplementary Table S2:** Primer sequences used for genes studied.

|  | **Forward primer** | **Reverse primer** |
| --- | --- | --- |
| IGF-1 | CTTGTTTCCTGCACTTCCTCT | CGCTGAAGCCTACAAAGTCA |
| IGFBP-1 | TGCCGGAGTTCCTAACTGTTGT | CCAGCGACTACGCGAACCT |
| IGFBP-3 | GGCCCAGCAGAAATATCAAA | TACCAGGGTCTCCAACAAGG |
| GHRH | ACTCTGGGTGTTCTTTGTGC | CCCTTGCTGCCTGTTCATGAT |
| Orexin | GAGGAGAGGGGAAAGTTAGG | TAGAGCCATATCCCTGCCC |
| GHSR1α | GAAGCCACCAGCTAAACTGC | GCTGCTGGTACTGAGCTCCT |
| Somatostatin | AGCCCAACCAGACAGAGAAC | CCTCATCTCGTCCTGCTCAG |
| NPY | CGCTCTATCCCTGCTCGTGT | GGTCTTCAAGCCTTGTTCTGG |
| AgRP | TGAAGAAGACAGCAGCAGACC | TGAAGAAGCGGCAGTAGCAC |
| PHD2 | CGAGCGAGCAAGAGCTAAAG | GGCAACTGAGAGGCTGTAGG |
| HIF1α | CCTACTATGTCGCTTTCTTGG | TGTATGGGAGCATTAACTTCAC |
| HIF2α | TTGCGGGGGTTGTAGATG | ACTTGGACGCTCTGCCTATG |
| β-actin | CCCGCGAGTACAACCTTCT | CGTCATCCATGGCGAACT |

IGF-1 – Insulin-like growth factor; IGFBP-1 – IGF binding protein 1, IGFBP3 – IGF binding protein 3; GHRH – Growth-hormone-releasing hormone; GHSR1α - growth hormone secretagogue receptors; NPY – Neuropeptide Y; – AgRP Agouti-related protein; PHD2 – prolyl hydroxylases; HIF 1α – Hypoxia-inducible factor 1-alpha.

**Supplementary References**

S1. Tarasiuk, A., Scharf, S. M., & Miller, M.J. Effect of chronic resistive loading on inspiratory muscles in rats. *J. Appl. Physiol*. **70,** 216–222 (1991).

S2. Tarasiuk, A., Berdugo-Boura, N., Troib, A., & Segev, Y. Role of GHRH in sleep and growth impairments induced by upper airway obstruction in rats*. Eur. Respir. J.* **38**, 870-877 (2011).

S3. Segev, Y., Berdugo-Boura, N., Porati, O., & Tarasiuk A. Upper airway loading induces growth retardation and change in local chondrocyte IGF-I expression is reversed by stimulation of GH release in juvenile rats. *J. Appl. Physiol.* **105,** 1602–1609 (2008).

S4. Tarasiuk, A., Levi, A., Berdugo-Boura, N., Yahalom, A., & Segev, Y. Role of orexin in respiratory and sleep homeostasis during upper airway obstruction in rats. *Sleep* **37,** 987-998 (2014).

S5. Schaub, C. D. *et al.* Effect of sleep/wake state on arterial blood pressure in genetically identical mice. *J. Appl. Phy*siol. **85**, 366-371 (1998).

S6. Everson, C. A., & Crowley, W. R. Reductions in circulating anabolic hormones induced by sustained sleep deprivation in rats. *Am. J. Physiol. Endocrinol. Metab.* **286,** E1060-E1070 (2004).

S7. Timofeeva, O. A., & Gordon, C. J., Changes in EEG power spectra and behavioral states in rats exposed to the acetylcholinesterase inhibitor chlorpyrifos and muscarinic agonist oxotremorine. *Brain Res*. **893,** 165-177 (2001).

S8. Obal, F. Jr., Payne, L., Kapás, L., Opp, M., & Krueger, J. M. Inhibition of growth hormone-releasing factor suppresses both sleep and growth hormone secretion in the rat. *Brain Res*. **557**, 149-153 (1991).

S9. Greenberg, H. E. *et al.* Effect of chronic resistive loading on ventilatory control in a rat model. *Am. J. Respir. Crit. Care. Med.* **152**, 666-676 (1995).

S10. Schneider, C. A., Rasband, W. S., & Eliceiri, K. W. NIH Image to ImageJ: 25 years of image analysis. *Nat. Methods* **9**, 671-675 (2012).
